# Development of In Situ Product Recovery (ISPR) System Using Amberlite IRA67 for Enhanced Biosynthesis of Hyaluronic Acid by *Streptococcus zooepidemicus*

**DOI:** 10.3390/life13020558

**Published:** 2023-02-16

**Authors:** Nur Imanina Abdullah Thaidi, Rosfarizan Mohamad, Helmi Wasoh, Mohammad Rizal Kapri, Ahmad Badruddin Ghazali, Joo Shun Tan, Leonardo Rios-Solis, Murni Halim

**Affiliations:** 1 Department of Bioprocess Technology, Faculty of Biotechnology and Biomolecular Sciences, Universiti Putra Malaysia, 43400 Serdang, Malaysia; 2 Bioprocessing and Biomanufacturing Research Complex, Universiti Putra Malaysia, 43400 Serdang, Malaysia; 3 Department of Oral Maxillofacial Surgery and Oral Diagnosis, Kulliyyah of Dentistry, International Islamic University Malaysia, 25200 Kuantan, Malaysia; 4 School of Industrial Technology, Universiti Sains Malaysia, 11800 Gelugor, Malaysia; 5 School of Natural and Environmental Sciences, Molecular Biology and Biotechnology Group, Newcastle University, Newcastle Upon Tyne NE1 7RU, UK; 6 School of Engineering, Institute for Bioengineering, University of Edinburgh, Edinburgh EH9 3JL, UK

**Keywords:** hyaluronic acid, extractive fermentation, in situ product recovery, ion-exchange resin, *Streptococcus zooepidemicus*

## Abstract

High broth viscosity due to the accumulation of hyaluronic acid (HA) causes a limited yield of HA. It is a major problem of HA production using *Streptococcus zooepidemicus*. Extractive fermentation via in situ product recovery (ISPR) was utilized to enhance the HA production. Resins from Amberlite: IRA400 Cl; IRA900 Cl; IRA410 Cl; IRA402 Cl; and IRA67 were tested for the HA adsorption. IRA67 showed high adsorption capacity on HA. The study of the adsorption via a 2 L stirred tank bioreactor of *S. zooepidemicus* fermentation was investigated to elucidate the adsorption of HA onto IRA67 in dispersed and integrated internal column systems. The application of a dispersed IRA67 improved the HA production compared to the fermentation without resin addition by 1.37-fold. The HA production was further improved by 1.36-fold with an internal column (3.928 g/L) over that obtained with dispersed IRA67. The cultivation with an internal column shows the highest reduction of viscosity value after the addition of IRA67 resin: from 58.8 to 23.7 (mPa·s), suggesting the most effective ISPR of HA. The improved biosynthesis of HA indicated that an extractive fermentation by ISPR adsorption is effective and may streamline the HA purification.

## 1. Introduction

Hyaluronic acid (HA) is a nonsulfated polysaccharide and has a molecular weight average of 10^5^ to 10^7^ Da [1,2]. The polymer is a linear glycosaminoglycan named mucopolysaccharide and composed of D-glucuronic acid and N-acetylglucosamine residues attached with b-1-3 and b-1-4 glycosidic bonds [2,3]. HA presents in many types of body parts of tissues and fluids and is one of the important parts of the extracellular matrix in the connective tissues [1]. The molecule was originally isolated from a bovine eye vitreous body in 1934 and was first described as a type of polysaccharide isolated from a vitreous body and umbilical cord blood, known as mucoitin sulfuric acid. The term hyaluronic acid is a combination of words “hyaloid” (vitreous body) and “uronic acid” [3]. 

HA has many biological functions and physicochemical properties, such as high water-retaining capability, mucoadhesive property, viscoelasticity, and biocompatibility [4]. At neutral pH, it attracts water and carries water from 1000 up to 10,000 times of its weight [2,5]. Due to its functions, HA is used in numerous applications, such as targeted drug delivery, orthopedics, ophthalmology, cancer therapy, rheumatology, dentistry, tissue engineering, and cosmetics [1,2,4,6,7]. HA comes from multiple sources through isolation and purification procedures, but it still has similar structures chemically. The majority of the extracted high molecular weight HA comes from animal origins, such as rooster comb and bovine vitreous humor. Nevertheless, the risk of inter-species viral infection and strict obligatory rules in the field of medicine and pharmaceuticals has changed the origin of HA from animal to microbial fermentation. In addition, the latter fermentation method is selected instead of the former extraction due to its added benefits of being more economical and has better purification efficiency [8].

*Streptococcus equi* subsp. *zooepidemicus* produce HA as a part of the cellular capsule [4]. It is a Gram-positive bacteria with aero-tolerance and catalase-negative characteristics. The streptococcus bacteria have spherical shapes and are commonly seen arranged in pairs or chains with capsules extracellularly surrounding them [8]. The main problem concerning HA production using microbial fermentation is the high broth viscosity that results in poor mixing and low oxygen mass transfer, which consequently reduce the yield of HA [9,10]. The presence of carboxylate groups in HA causes it to be negatively charged, and its anionic nature enables it to bind to a significant quantity of water to create a viscous gel [11]. The accumulation of HA will increase the broth viscosity during HA biosynthesis. Furthermore, the efficiency of the agitation rate is vitally affected by the non-Newtonian fluid and high viscosity of broth as HA is accumulated throughout the fermentation period [12]. The conventional approaches to increase the oxygen mass transfer rate have been directed towards utilizing strategies of increasing agitation or aeration rate [13], maintaining the dissolved oxygen in the media [14], improving the bioreactor design [15], optimization with medium formulation and impeller [16], and the application of oxygen vector [17], each with their limitations and little improvements.

Another way to improve fermentation is by an extractive fermentation employing suitable adsorbents. Generally, this method depends on product and by-product inhibition. For an example, activated carbon was employed as an adsorbent in a study by Gao et al. (2011) on an extractive fermentation of lactic acid [18]. The use of activated carbon has removed the inhibitory action of lactic acid. Subsequently, the productivity and yield of lactic acid has been increased in this pH-uncontrolled fermentation. In situ product removal (ISPR) employing various types of adsorbent resins has been widely used in bioseparation. From a downstream processing perspective, applying ISPR to biological processes, such as fermentative production and a bioconversion process, may reduce the number of subsequent downstream processing operations, and this too may result in a reduction of product losses [19].

The simplest approach to implementing ISPR is to introduce the adsorbent directly into the stirred tank bioreactor (STR) in a dispersed condition where it remains throughout the process. At the end of the batch operation, the adsorbent can be recovered, and the by-product compound is desorbed. In this configuration, the mass of adsorbent added to the STR must be sufficient so that the product produced (i.e., HA) can be fully recovered and the broth viscosity can be maintained below the threshold throughout the fermentation process. The alternative approach to implementing ISPR is to continuously recirculate the bioconversion medium through an internal column-packed bed of adsorbent [20]. The advantages of this approach are that the adsorbent is separated inside the STR and it is easy to collect the adsorbent and recover the HA product at the end of the process by elution of the adsorption column.

The use of resin as HA adsorbent material can be influenced by a number of factors, such as its biocompatibility with microbes, regenerability, and types of the resin [20]. For instance, resin chosen as an HA adsorbent should have high capacity and selectivity for HA rather than water, as well as other substrates, such as glucose, acetic acid, alanine, and tryptone that may be present [21]. Different types of resin show different preferences or selectivity for certain ions from nutrients [22]. In addition, different types of resin also have different total exchange capacities which might affect the bond formation between ions and the efficiency of adsorption. According to several articles in the literature and product data sheets, weak base resins are more suitable to be applied in the adsorption of organic acids compared to strong base resins owing to their high oxidative resistance and less organic fouling [23]. Resin with a cross-linked acrylic gel matrix is more hydrophilic and has high selectivity for most organic acids compared to resin with a styrene matrix [19].

The application of ISPR can streamline the procedures of fermentation production and bioconversion process as well as reduce product loss [19]. Product recovery (i.e., HA) by using adsorptive resins during the fermentation stage can reduce the overall required steps, as the conventional HA purification steps include depth filtration, diafiltration, selective adsorption, and finally, a solidification and drying process [24]. To our knowledge, there has been no study reported, to date, on applying adsorbent resin for ISPR during HA fermentation, while only one piece of data has been patented on the utilization of aromatic adsorption resins for the purification of HA after the fermentation step [25]. The purpose of this study was to evaluate the efficiency of dispersed and integrated internal column systems of Amberlite IRA67 ion-exchange resin for ISPR of HA and enhancement of HA production by *S. zooepidemicus*.

## 2. Materials and Methods

### 2.1. Glycerol Stock of Streptococcus zooepidemicus

The bacteria strain, *S. zooepidemicus* HA-116 used in this study was obtained from the American Type Culture Collection (ATCC) (Rockville, MD, USA). The strain was kept at −30 °C in 30% (*v*/*v*) glycerol (Sigma, St. Louis, MI, USA). The solid medium, comprised of tryptic soy agar (Merck, Germany) with the addition of 5% (*v*/*v*) horse blood (Veterinary Clinic, Faculty of Veterinary Medicine, UPM), was used for the stock culture. The culture was incubated for 24 h at a temperature of 37 °C.

### 2.2. Ion-Exchange Resins and HA Adsorption Capacity

Five commercially available polymeric resins (Sigma, USA) called Amberlite IRA67 Freebase (IRA67), IRA410 Cl, IRA900 Cl, IRA402 Cl, and IRA400 were screened to determine the most suitable ion-exchange resin for ISPR of HA. The resin preparation procedure was adopted by Luongo et al. 2018 [26], to improve the performance of the resin. The pre-treatment of the resins included washing steps: (i) 1 M of NaOH solution, (ii) distilled water, (iii) 1 M of HCl solution, (iv) distilled water, (v) 1 M NaOH solution, and finally, (vi) washed with distilled water until pH 7.

For sterilization, resins were exposed to a UV light source in a laminar flow cabinet for one hour at room temperature, prior to each experiment. Adsorbent dosage strongly affected the sorption capacity. For the adsorption study, the resin was individually added into a 15 mL Falcon tube containing 10 mL of HA at a prefixed concentration. The adsorption experiment was carried out for the resin dose of 5–25 g/L. Shaker agitation was done until sorption reached equilibrium at 250 rev/min for 24 h. Then, the resin and solution were centrifuged at 10,000× *g* for 10 min to separate the resin from the solution. The remaining HA left in the solution was determined by the supernatants.

The HA per unit amount of resin (*q_e_*) and percentage of HA adsorbed (%) were calculated using Equations (1) and (2) below:

*q_e_* = (*C*_0_ − *C_t_*)*V*/*m* (1)

% HA adsorbed = (*C*_0_ − *C_t_*)100/*C*_0_ (2)

where *q_e_* is the amount of solute (HA) adsorbed per unit amount of adsorbent (resin) at time *t* (mg/g), *C*_0_ is the initial HA concentration (mg/L), *C_t_* is the residual HA concentration in solution at time *t* (mg/L), *V* is the volume of the solution (L), and *m* is the mass of resin (g).

Resins can be regenerated and used again by using distilled water and regenerating with 1M NaOH to elute the HA that was adsorbed at an ambient temperature. The wet resin was initially dried, and sterilized by the UV light. Equation (3) was used to calculate the total amount of HA produced from the fermentation:

A [g/L] = B [g/L] + C [g/L](3)

where, A is the total HA, B is the equilibrium HA in the broth culture (supernatant), and C is the HA eluted from the adsorbent resin.

For the preliminary study, 50 g/L of the Amberlite IRA67 resin was added into different growth phases of *S. zooepidemicus* fermentation media to determine the most suitable growth phase for the addition of the resin into the media. Fermentations of *S. zooepidemicus* were performed in 250 mL Erlenmeyer shake flasks containing 100 mL fermentation media. Furthermore, different dosages of IRA67 concentration (0–60 g/L) were introduced into the cultivation media to further study the effect of different resin dosages on HA biosynthesis produced by *S. zooepidemicus* fermentation. The IRA67 resins were sterilized using UV light before being introduced into the flasks during inoculation. The flasks were inoculated with 10% (*v*/*v*) inoculum and incubated at 37 °C at 250 rev/min for 24 h without pH control.

### 2.3. Cultivation of S. zooepidemicus and Experimental Design

#### 2.3.1. Inoculum

Inoculum preparation was done by subculturing the glycerol stock culture for 12 h in a 250 mL Erlenmeyer flask containing 100 mL of tryptic soy broth (Merck, Darmstadt, Germany) [16]. The flask was incubated in a rotary shaker at 250 rev/min at 37 °C. The cells were harvested at mid-exponential to be used as an inoculum. This was to avoid harvesting cells at late exponential or early stationary phase (when optical density was in the range of 0.6–0.8) to ensure the inoculum cells were in an active state and quickly adapt to a fresh media environment. The optical density was read at 600 nm using a spectrophotometer (Biochrom S12 Libra, Cambridgeshire, UK).

#### 2.3.2. Batch Fermentation of S. zooepidemicus

**Figure 1:**
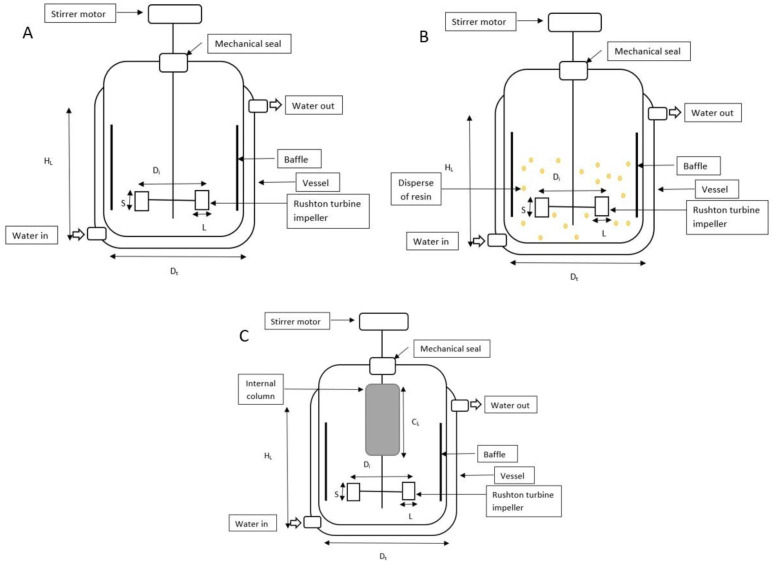
Schematic diagram, dimensions, and operating variables for 2-L STR. (**A**) The bioreactor (without resin); (**B**) 2-L STR with disperse system; (**C**) 2-L STR integrated with internal column system.

The experiments to investigate the influence of extractive fermentation with an ion-exchange resin in HA biosynthesis were carried out in batch fermentations using a 2-L STR (Biostat, B. Braun Biotech International, Germany). The fermentation medium containing (in g/L): glucose 50, tryptone 15, yeast extract 5, KH_2_PO_4_ 2, K_2_HPO_4_ 2, and MgSO_4_. 7H_2_O 0.5 was sterilized at 121 °C for 15 min [16]. Fermentations were carried out for at least a duplicate and the mean value of each experiment was obtained. The STR was equipped with six-bladed disc Rushton turbine impellers, pH, temperature, and dissolved oxygen controllers. The internal column was aseptically packed with the UV-sterilized Amberlite IRA67 (50 g/L) and attached to the 2-L STR (Figure 1C).

The oxygen probe response to variation of the dissolved oxygen concentration is assumed to be rapid and instantaneous. The sterilized 2-L STR containing 900 mL of medium was inoculated with 100 mL of the inoculum culture (10%, *v*/*v*). The bioreactor’s temperature was kept at 37 °C, while the culture’s initial pH was adjusted to 7 and maintained with 3 M NaOH. Every experiment was run at 1 vvm aeration. The batch HA fermentation’s agitation rate was kept at 200 rev/min. The configuration of the 2-L STR was described in Figure 1 and Table 1.

### 2.4. Analytical Methods

Sample for cell concentration, HA, lactic acid (LA), and glucose concentrations determination was withdrawn during fermentation for analysis. The methods for cell concentration, HA, LA, and glucose determination were briefly described below.

#### 2.4.1. Cell Concentration

Cell concentration (gram of dry cell weight (DCW) per liter) was determined based on the optical density (OD) measurement at a 600 nm spectrophotometer. The OD value (1 mL) was initially measured and transferred into a 1.5 mL centrifuge tube. The samples were centrifuged at 3000× *g* for 10 min and the supernatants were collected to use for other determinations. The pellet was washed with 0.9 % saline solution and redissolved into 1 mL of sterile distilled water. The OD of the washed pellet was read under 600 nm. For the DCW of the sample, a known volume of the sample was filtered through a 0.45 µm cellulose nitrate membrane filter under vacuum suction. After being thoroughly washed with sterile distilled water, the cell paste retained was dried in an oven set at 100 °C and weighed (until constant dry weight was achieved). The correlation between DCW and OD was estimated from the batch experiments as described by the Equation (4):

g DCW = 0.6072 × (OD) + 0.0495(4)

#### 2.4.2. HA Concentration

The cells from fermentation broth were removed by centrifugation (10,000× *g* for 20 min) and the supernatant was subjected to a precipitation method. The HA was precipitated with 2 volumes of absolute ethanol by refrigeration at 4 °C for 2 h. The precipitate was then collected by centrifugation (3000× *g* for 20 min) and subsequently re-dissolved with distilled water (Volume of distilled water = volume of sample). The carbazole method was used to quantify HA [27]. After the purplish-pink color was formed, the adsorption of the sample was measured at 530 nm using a spectrophotometer. HA from *S. zooepidemicus* (Sigma-Aldrich, Petaling Jaya, Malaysia) was used to prepare the standard calibration curve (absorbance versus HA concentration)**.**

#### 2.4.3. Lactic Acid Assay

The lactic acid (LA) analysis was analyzed using the l- lactate assay kit (Megazyme, Ireland). Briefly, a volume of 0.1 mL of supernatant was added to the solution containing distilled water (1.5 mL), buffer (0.5 mL), NAD^+^/PVP (0.1 mL), and D-GPT (0.02 mL). The solution was mixed and read at 340 nm with a spectrophotometer. The mixture was mixed with L-LDH solution (0.02 mL) for 10 min and read for the absorbance (340 nm) at 5 min intervals until the absorbance remained the same. The concentration of LA (c) was calculated as follows:

c = [(V × MW)/(ε × d × v)] × ΔAL-lactic acid [g/L](5)

where:

V = final volume [mL];
MW = molecular weight of L-lactic acid [g/mol];
ε = extinction coefficient of NADH at 340 nm = 6300 [l × mol^−1^ × cm^−1^];
d = light path [cm];
v = sample volume [mL].

#### 2.4.4. Glucose Assay

The glucose analysis was analyzed using the D-glucose assay analysis kit (Megazyme, Wicklow, Ireland). Briefly, the sample was centrifuged at 3000× *g* for 10 min to remove the cells. The 3.0 mL of GOPOD Reagent was added into 0.1 mL of a sample solution containing D-glucose. The mixture was then incubated at a temperature between 40 and 50 °C for 20 min. Absorbances were read at 510 nm against the reagent blank to obtain Δsample and ΔD-glucose standard. The glucose can be calculated as Equation (6):

Glucose (µg/0.1 mL) = (Absorbance sample/Absorbance glucose standard [0.1 mL]) × 100(6)

#### 2.4.5. Scanning Electron Microscope

The morphology of *S. zooepidemicus* and the surface area of anion-exchange resins were investigated under the scanning electron microscope (SEM) (JSM-IT 100, Jeol, Akishima, Japan). The method was adopted from Othman et al. (2018) [20] with slight modifications. The samples were prepared by fixation that was done with 4% (*v*/*v*) glutaraldehyde buffer for 12 h at 4 °C. The samples were then washed with 0.1 M sodium cacodylate buffer for 10 min. Next, the samples were fixed with 1% (*w*/*v*) osmium tetroxide for 2 h at 4 °C and washed once again. Then, the samples were dehydrated with increasing serial concentrations of acetone. The samples were placed in the sputter coater chamber after being mounted on an aluminum stub with a double stick of carbon tape. The samples were subsequently coated with a thin layer of metal gold/palladium (40–60 nm) and magnified at 2000× and 5000×.

#### 2.4.6. Viscosity Analysis

Viscosity is the measure of a fluid’s resistance to flow (shear stress) at a given temperature. In this study, the viscosity value of the fermentation broth of *S. zooepidemicus* was measured because HA accumulation affects the viscosity of the cultivation media. Briefly, the viscosity of the media (40 mL) was measured using a viscometer (Vibro SV-10, A&D, Tokyo, Japan) at room temperature.

### 2.5. Statistical Analysis

SPSS software (Version 20) and Microsoft Excel (Office 365) were used for the statistical analysis of data. The reported results are the mean of at least duplicates and are shown as mean value standard error. To determine significant differences (*p* < 0.05) between samples, the unpaired T-test and one-way analysis of variance (one-way ANOVA) were used.

## 3. Results and Discussion

### 3.1. Ion-Exchange Resins Adsorption Capacity of HA

**Figure 2:**
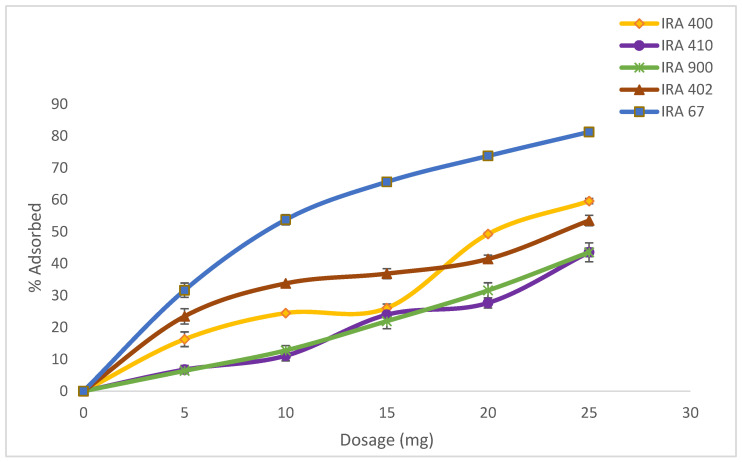
Effect of resin adsorbents concentration range from 0 to 25 g/L in the adsorption of HA. Error bars represent the standard deviations of the mean of experiment (*n* = 3).

Adsorption experiments of various resin dosages starting from 5 to 25 g/L were carried out (Figure 2). Four strong base anion resins (Amberlite IRA400 Cl, Amberlite IRA410 Cl, Amberlite900 Cl, and Amberlite402 Cl) and one weak base anion resin (Amberlite IRA67) were screened for the highest uptake capacity of HA based on HA percentage adsorption capacity. The ion-exchange resins can be identified based on the supporter matrix and the exchange capacity for organic acids [28]. Table 2 shows the comparison between all the resins in terms of percentage adsorbed. From all the resins tested, three resins gave HA adsorption of more than 50%. The highest adsorption of HA was from resin Amberlite IRA67 (81.80 ± 0.56%), followed by IRA400 Cl, and IRA402 Cl, respectively. When the resin selections are analyzed, it was observed that the base resins, such as IRA67, are more efficient than the strong base resins for HA adsorption. This observation is consistent with prior research indicating that weak base resins outperform strong base resins for organic acid separations [29].

The method of preparation and pretreatment of the resin can influence the adsorption capacity of the resin. IRA67 has been pretreated with 1 M NaOH and 1 M HCl to form a pH closer to 7. Furthermore, the adsorption capacity of weakly basic ion-exchange Amberlite IRA67 will decrease with increasing pH values [30]. Phenolic compounds and organic acids gave higher adsorption capacity when the Amberlite IRA67 was closer to a neutral pH regime [31].

The chemically altered resin can modify the interaction strength between the solutes and surface of the adsorbent and improve the adsorption capacity even with a smaller surface [32]. For example, the chemically modified Amberlite XAD-2 resin was showed to possess higher adsorption capacity for aspartame [33]. The chloromethylated XAD-2 resin, with a smaller surface area of 143 m^2^/g, can adsorb 25% more aspartame than the unmodified XAD-2 resin, which has a higher surface area of 312 m^2^/g. In addition, Wang et al. (2010), reported a comparable 95% concentration reduction (from an initial amount of 80 mg/L) for vanillin and syringaldehyde after the adsorption by D101 resin, which has a surface area value of 400–550 m^2^/g within 120 min [34].

The resin’s biocompatibility with microorganisms is another important factor to be chosen as an HA adsorbent [35]. Most of the ion-exchange resins show no toxic characteristics to microorganisms, and therefore they can directly be included in the bioreactor [21]. Amberlite IRA67 has a matrix of cross-linked acrylic gel, and it has higher water affinity than styrene resins [20]. Meanwhile, styrene resins have many aromatic rings, making them more hydrophobic [20]. As a result, Amberlite IRA67 was chosen and used in the subsequent experiments to investigate its potential in HA extractive fermentations.

### 3.2. Effect of Different Dosages of Amberlite IRA67 on HA Production by S. zooepidemicus

The total HA concentration with the addition of IRA67 (50 g/L) from the beginning of cultivation (lag phase), late in the exponential phase, and during the stationary phase was shown in Table 3 for the preliminary study. This study was conducted to determine the most suitable growth phase for the addition of resin into *S. zooepidemicus* fermentation media. The total HA produced from the fermentation media with the addition of resin during the stationary phase (1.174 g/L) was higher compared to the addition of resin late in the exponential phase (0.537 g/L) and the lag phase (0.148 g/L) of *S. zooepidemicus* fermentation. Furthermore, several studies have also suggested introducing the ion-exchange resin during the stationary phase in fermentation [36,37,38]. Hence, the addition of the resin into *S. zooepidemicus* fermentation during the stationary phase was employed in the further experimental study of IRA67 on HA production.

Table 4 shows the effect of different Amberlite IRA67 (IRA67) dosages (ranging from 0 to 60 g/L) on the HA production by *S. zooepidemicus*. The best dosage of IRA67 resin was found to be 50 g/L, with the highest total HA production (1.174 ± 0.003 g/L). Some of the main reasons for variation in the dosage amount of adsorption are acidic/basic characteristics, surface area, pretreatment, particle size, and ionic form of the resin. Adsorbent dosage strongly affected the sorption capacity [32]. Based on the result of this study, with the fixed HA concentration, the percentage of HA adsorbed increased with the increasing weight of the IRA67 resin. This was due to increased availability of active sites or surface area at greater adsorbent concentrations [32]. The low dosage of IRA67 causes low adsorption capacity due to limited adsorption sites that have been fully occupied by adsorbates [39]. This will then lead to a limit on the available sites for the adsorption of HA.

Although increasing the dosage concentration of IRA67 increased the adsorption of HA, the addition of more than 50 g/L of IRA67 showed a gradual decrease in the total HA concentration. At the highest loading concentration of 60 g/L, the total HA concentration was reduced to 1.107 g/L. Ion-exchange resins typically have different affinity levels towards nutrients and other compounds that are available in the culture. This could be why there are different inhibitory effects toward cells for each type of ion-exchange resin [40,41]. The results of this study are consistent with those of Pradhan et al. (2017), who found that other organic substances present in the media, such as glucose and LA, compete for adsorption [21]. This, consequently, reduces the amount of HA adsorbed on the ion exchange resin sites and, as a result, lowers the total yield of HA. The adsorption capacity of Amberlite IRA67 for HA may be reduced by competitive adsorption when it occurs. Luongo et al. (2019) stipulated that when other anion components in the culture are present, they may compete with the target compound for adsorption on the anion exchange resin sites [26]. The lower total HA concentration observed with a higher dosage of over 50 g/L of IRA67 resin could also be attributed to shear stress caused by such a high resin concentration [20].

### 3.3. Batch Fermentation of S. zooepidemicus

**Figure 3:**
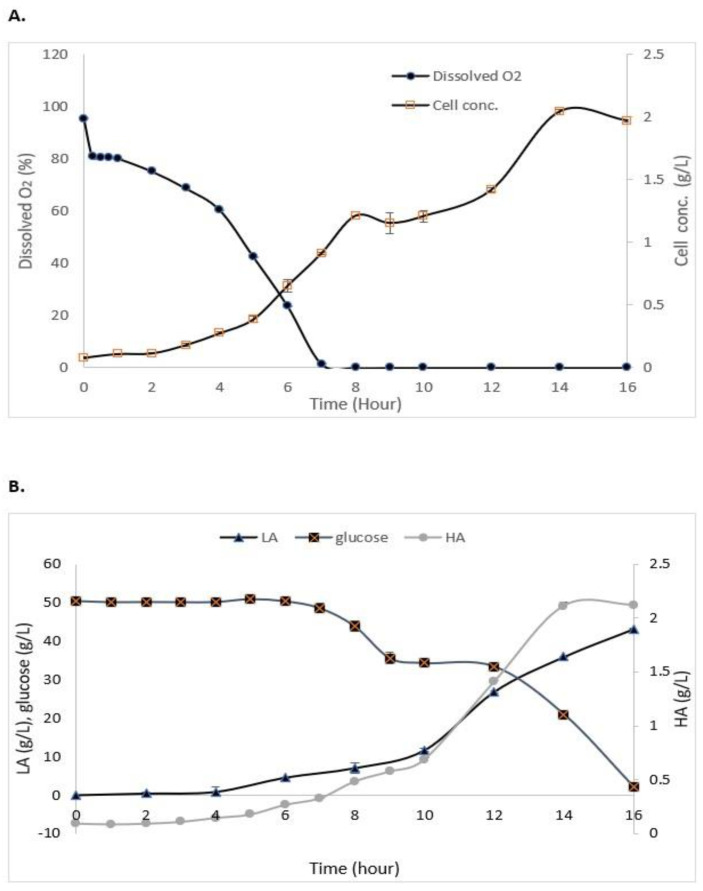
Experimental data from the batch fermentation of *S. zooepidemicus* in STR without the addition of Amberlite IRA67 resin. (**A**) Dissolved oxygen and cell concentration; and (**B**) glucose consumption and HA and LA productions. Error bars represent the standard deviations of the mean of experiment (*n* = 3).

As shown in Figure 3A, the maximum growth of *S. zooepidemicus* in the STR without the addition of IRA67 resin was obtained at 14 h (stationary phase) of cultivation with 2.03 g/L. The log phase or exponential phase was started at 5 h of cultivation and the growth started to decrease at 15 h, and this indicates the cells had entered a death phase. The air saturation (dissolved oxygen) gradually decreased as the time of cultivation increased. Glucose consumption, HA, and LA production are represented in Figure 3B. The initial concentration of glucose is 50 g/L. The glucose concentration decreased as the time of cultivation increased with 2.057 ± 0.15 g/L at the end of the fermentation. HA concentration was increased when the fermentation entered 4 h of cultivation, and the highest HA concentration (2.11 ± 0.035 g/L) was measured at 14 h. The LA concentration was drastically increased towards the end of cultivation to reach 43.2 ± 0.08 g/L.

**Figure 4:**
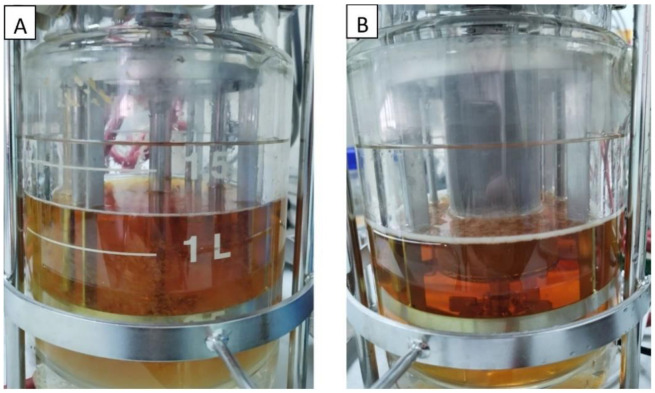
Fermentation media of *S. zooepidemicus* in 2-L STR with Amberlite IRA67 resin. (**A**) Disperse; (**B**) internal column.

The batch fermentations with the addition of IRA67 resin in dispersed and internal column systems were initially started with the cultivation of *S. zooepidemicus* without the presence of IRA67 resin. Only after the cultivation reached 14 h (the highest HA production as observed in the growth profile study) was 50 g/L of IRA67 resin added to the fermentation media in the 2-L STR (Figure 4).

**Figure 5:**
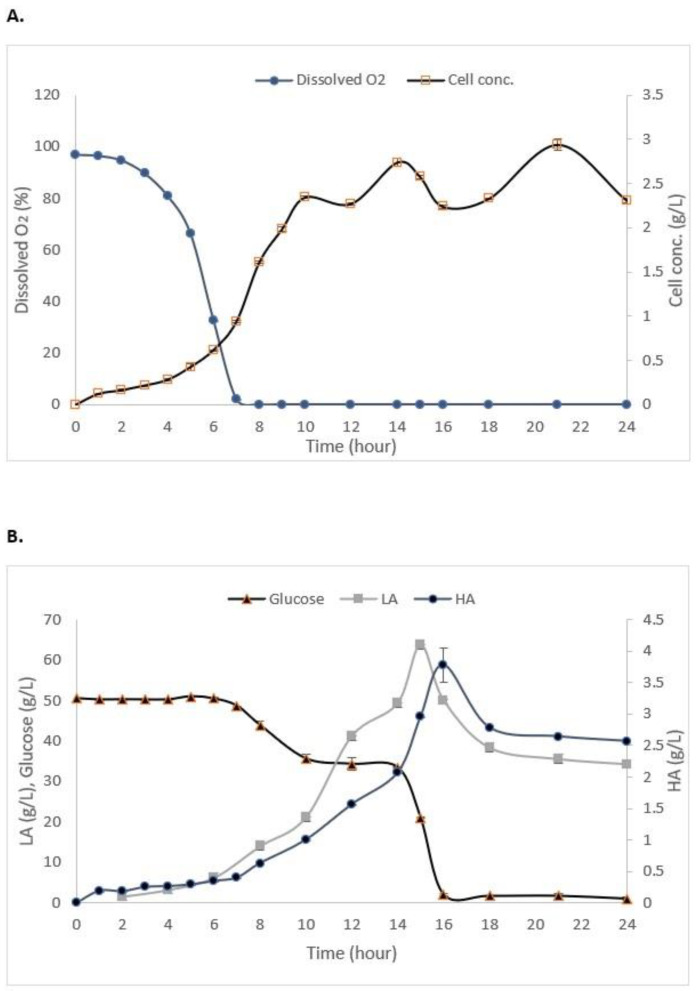
Experimental data from the batch fermentation of *S. zooepidemicus* in in the STR integrated with the internal column of IRA67. (**A**) Dissolved oxygen and cell concentration; and (**B**) glucose consumption and HA and LA productions. Error bars represent the standard deviations of the mean of experiment (*n* = 3).

The cell concentrations of *S. zooepidemicus* in the STR with dispersed resin (2.47 ± 0.06 g/L) (Table 5) and the internal column (2.934 ± 0.059 g/L) (Figure 5A) were found to be significantly higher than that of the control cultivation without the addition of resin (2.03 ± 0.09 g/L). One of the strategies for enhancing HA production is by developing an effective process for high-cell-density cultivation [42]. The air saturation (dissolved oxygen) in both dispersed (Table 5) and the internal column (Figure 5A) gradually decreased as the time of cultivation increased. The oxygen fell to zero during the exponential phase and remained at the zero level until the end of the fermentation, which is similar to the cultivation without resin. This occurred due to the rheological characteristics of the accumulated HA in the fermentation broth [43].

There were significant differences in the HA and LA concentrations in the 2-L STR fermentation between control (no resin), with the dispersed and internal column IRA67 resin. The total HA produced from the fermentation of S. *zooepidemicus* inside 2-L STR with the addition of IRA67 resin in dispersed system (2.872 g/L) was higher compared to the total HA produced by the fermentation without the addition of resin, which is equivalent to 1.37-fold. The HA production was further improved by 1.36-fold and 1.87-fold with an internal column (3.928 g/L) over that obtained with dispersed IRA67 resin and control fermentation without the addition of resin, respectively. The increased yield may be due to suitable environmental conditions created by this method. The surface of the resin adsorbent packed in the column can be increased because the use of an internal column allows the fluidization of porous adsorbent resin; therefore, more adsorbates can be adsorbed [44]. To date, columnar resins have been used to enhance multiple product biosynthesis, overcome the product inhibitory effect, and streamline the procedure for product recovery. The recovery of LA from *P. acidilactici* [20], the culture of *L. plantarum* [45], the enhanced production of periplasmic interferon alpha-2b by *E. coli* using an anion-exchange resin for ISPR of acetic acid [46], and the use of ion exchange resins for purification of LA produced from a cassava syrup fermentation [47] are some examples from the literature.

After the addition of IRA67 resin onto STR during the stationary phase (at 14 h), the LA concentrations have been observed to decrease over time for both dispersed (from 55.629 g/L to 42.789 g/L) and internal column system (from 63.69 g/L to 34.32 g/L). This finding is beneficial, as the LA accumulation may reach a concentration that inhibits cell growth and HA production [48]. *Streptococcus* is known to produce a high amount of LA, and hence consume a high volume of NaOH, which then increases the viscosity and lowers the mixing of the broth [49]. The high HA concentration can be obtained by reducing the priority of the glycolytic pathway and optimizing the enzyme metabolic pathway [50,51]. The synthesis of unwanted metabolites (e.g., L-lactate) was restrained in order to boost the generation of intermediate metabolites essential for HA synthesis [52,53]. The glucose concentrations for both 2-L STR with dispersed (1.27 ± 0.78 g/L) (Table 5) and internal column (0.957 ± 0.41) (Figure 5B) were decreased at the end of fermentation, which was similar to the control fermentation (no resin) (2.05 ± 0.12 g/L). Glucose was one of the important nutrients for enhancing HA production [54]. However, *Streptococcus* are lactic acid bacteria that consumed glucose and convert the glucose into lactate by glycolysis [55]. Beside lactate, other compounds, such as acetate and formate, were also produced and accumulated during *Streptococcus* fermentation, causing the decrease of pH of the cultures, and thereby inhibiting the microbial growth and HA production [56,57]. This can be seen from the data shown in the control fermentation, which has consumed almost all glucose, but the cell growth and HA concentration were lower compared to the system with the addition of the Amberlite IRA67. The addition of IRA67 in the STR with *S. zooepidemicus* fermentation media has been shown to reduce LA concentration, indicating decreasing lactate production, and therefore, decreasing glycolysis [58].

### 3.4. Scanning Electron Microscope of Fermentation Broth and the IRA67 Resin

**Figure 6:**
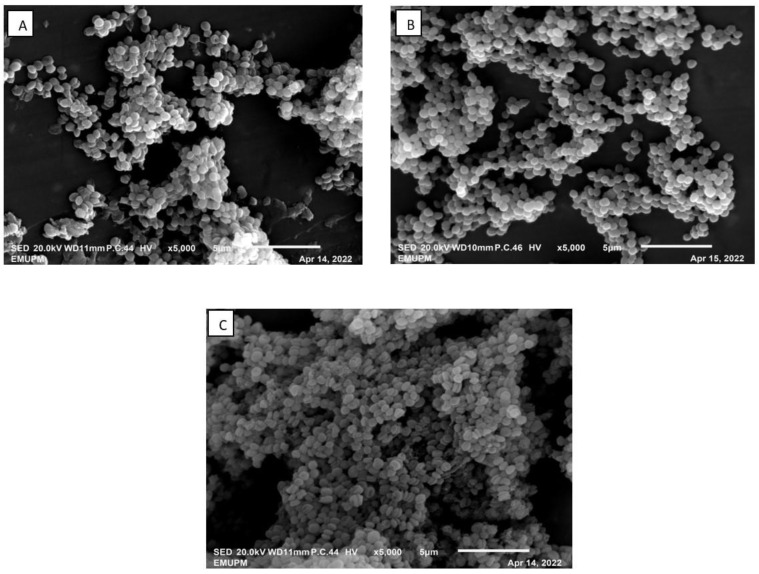
SEM photograph (magnification of ×5000) of *S. zooepidemicus* cultivated in 2-L STR. (**A**) Control (no resin); (**B**) dispersed IRA67 resin; (**C**) IRA67 resin packed in an internal column.

The samples of broth cultivation and IRA67 resin from the STR integrated with dispersed and internal column system were examined under a scanning electron microscope (SEM). As shown in Figure 6, the morphology of *S. zooepidemicus* under SEM showed that the strain was cocci in shape with single, pair, and chain. The growth morphology of *S. zooepidemicus* was similar when compared the control fermentation with the extractive fermentations, either dispersed resin or internal column system. This finding was in line with the study by Othman et al. (2018), where the morphology of *P. acidilactici* in the fermentation broth was observed to be similar between the control (without resin) and the fermentation with the addition of IRA67 resin [20]. Tan et al. (2011), also showed that there was no variation in *E. coli* morphology between fermentations with different types of resins (WA30 and M43) and the control fermentation (without resin) [28].

**Figure 7:**
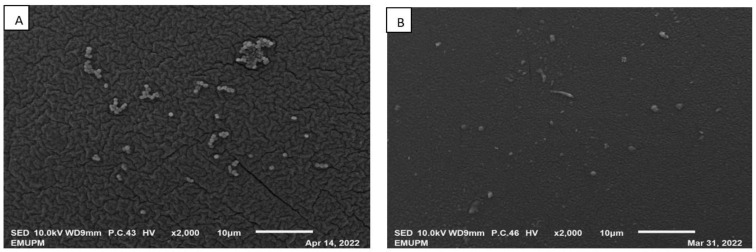
SEM photographs (magnification of ×2000) of the surface structure of IRA67 from fermentation in 2-L STR. (**A**) Dispersed IRA67; (**B**) internal column of IRA67.

As depicted in Figure 7, when the IRA67 resin was collected from the broth at the end of fermentation and examined under SEM, the surface of IRA67 was largely consistent and smooth [59]. It is evident that some of the molecules interact with the resin’s active areas. There was also a small number of cells that were seen attached to the resin surface. Nevertheless, very minimal cell agglomeration was detected on the resin surface, particularly for the resins taken from the interior column. Likewise, the previous report on the in situ addition of anion-exchange resins (WA30 and M43) for enhanced *E. coli* growth and expression of periplasmic human interferon-α2b, also observed that no cell agglomeration can be found beside some cells attached to the surface of the resin area [28]. The use of 2-L STR integrated with an internal column for an adsorption process allows the cell to move freely through the column, whilst the target by-product was captured [60].

Amberlite IRA67 with a concave surface and scratch was observed from the fermentation with dispersed resin condition (Figure 7A). Whereas, smooth IRA67 resin surfaces were observed from the fermentation with an internal column bioreactor system (Figure 7B). This finding may stipulate that the internal column reduces the resin collision, which then reduces the shear force. This observation is in agreement with the study by Tan et al. (2013) that reported on the fermentation of *E. coli* with the addition of ion-exchange resin Diaion WA30, to achieve a higher synthesis of periplasmic interferon alpha-2b [46]. They also observed a concave surface for the resins applied as a dispersion method. The concave surface resins (for the resins in the dispersed system) may be caused by the direct shear force from the impeller and increasing rate of resin collision, which affects the stability and efficacy of the resins. The internal column system allows the resins to adsorb the target product by being entrapped inside the column with no direct contact with the impeller [44]. Furthermore, the life cycle for IRA67 is higher in the internal column than in the dispersed system, as the letter, the concave-shaped surface, may clog the resin pores and cause fouling of the resin [61].

### 3.5. Viscosity Analysis on Fermentation Media

Synthesis of some biopolymers from the microbial fermentations is commonly linked with high viscosity that results in poor mixing and low oxygen mass transfer, and consequently, reduces the product yield [62]. For HA biosynthesis, mass and energy transfer become a particular concern due to the high viscosity of the HA product [63]. HA comprises carboxylate groups, which give it a negative charge [11] and allows it to bind to a large amount of water creating an extremely viscous gel. In ISPR configuration, the mass of adsorbent added to the STR must be sufficient so that the product produced (i.e., HA) can be fully recovered and the broth viscosity can be maintained below the threshold throughout the fermentation process [64].

In this study, the viscosity value of *S. zooepidemicus* fermentation media was measured to evaluate the effect the ISPR system has on controlling the viscosity of the broth throughout the extractive fermentation. The initial viscosity value of the fresh fermentation medium (before the inoculation of *S. zooepidemicus*) was 2.50 mPa·s.

Table 6 shows the viscosity value of the fermentation broth of *S. zooepidemicus* in different types of cultivation of resin systems. The cultivation media from 2-L STR with dispersed (40.0 mPa·s) and internal column (23.7 mPa·s) showed a reduction of viscosity value after the introduction of the resin compared to the control fermentation (without resin) (60.5 mPa·s). The viscosity from fermentation integrated with the internal column shows the highest reduction of viscosity value after the addition of IRA67 resin, from 58.8 mPa·s to 23.7 mPa·s, suggesting the most effective adsorption of HA accumulation. There were significant differences (*p* < 0.05) in viscosity values between the broth from 2-L STR of control fermentation media, dispersed, and internal column of IRA67. Some other studies also have been shown to successfully reduce the viscosity with the application of adsorbents. For example, the application of granule-activated carbon and increasing the temperature to an anaerobic digestion system has been shown to reduce the viscosity and, simultaneously, improve the material and energy transfer [65]. Another study by Yu et al. (2022), showed that the VR-1 resin has good viscosity reduction ability for heavy oil [64].

## 4. Conclusions

ISPR addition of the selected resin during the fermentation of the culture can lead to a higher production of HA by *S. zooepidemicus*. When tested with five different anion exchange resins, Amberlite IRA67 at a 50 g/L concentration showed the highest production of HA using *S. zooepidemicus*. The selection of the internal column method also showed a higher production of HA by 1.36-fold and 1.87-fold when compared to the dispersed system and control fermentation (without resin), respectively, by solving the resin collision and high shear force problems in the dispersed method. Ion-exchange resins have also demonstrated the ability to lower the viscosity of the fermentation broth by adsorbing some of the accumulated HA. Therefore, the improvement of biosynthesis of HA indicated the effectiveness of an extractive fermentation by ISPR ion-exchange adsorption that may also simplify the HA purification steps.

## Figures and Tables

**Table 1:** Dimension and variable value of 2-L STR with Rushton turbine impeller.

Dimension and Variables	Value
D_i_ (m)	0.083
D_t_ (m)	0.130
H_L_ (m)	0.120
S (m)	0.010
D_i_/D_t_ (m)	0.638
L (M)	0.015
C_L_ (m)	0.120
Speed (rev/min)	200
Airflow rate (vvm)	1.00

**Table 2:** Amount of HA adsorbed and the characteristics of the resin.

Type of Resin (Amberlite)	Amount of HA Adsorbed (%)	Characteristic	Particle Size (mm)	Ionic Form
IRA400 Cl	58.73 ± 0.84	Strong base	0.6–0.75	Cl^-^
IRA900 Cl	42.19 ± 1.35	Strong base	0.6–0.75	Cl^-^
IRA410 Cl	40.58 ± 2.96	Strong base	0.64–0.8	Cl^-^
IRA402 Cl	51.85 ± 1.63	Strong base	0.6–0.75	Cl-
IRA67	81.80 ± 0.56	Weak base	0.5–0.75	Free base

**Table 3:** The concentration of total HA with the addition of IRA67 at three different growth phases of *S. zooepidemicus*.

Growth Phase	HA Concentration (g/L)
Lag	0.148 ± 0.80
Late exponential	0.537 ± 1.23
Stationary	1.174 ± 0.003

**Table 4:** Effect of different IRA67 dosages on the HA production by *S. zooepidemicus* fermentation media.

IRA67 Dosage (g/L)	HA in Media (g/L)	HA Eluted (g/L)	Total HA (g/L)
0	0.312 ± 0.002	-	0.312 ± 0.002
15	0.236 ± 0.001	0.232 ± 0.003	0.468 ± 0.002
25	0.285 ± 0.001	0.296 ± 0.002	0.581 ± 0.001
35	0.323 ± 0.001	0.298 ± 0.002	0.621 ± 0.002
45	0.564 ± 0.018	0.312 ± 0.003	0.876 ± 0.007
50	0.356 ± 0.001	0.818 ± 0.004	1.174 ± 0.003
55	0.287 ± 0.010	0.857 ± 0.005	1.144 ± 0.006
60	0.404 ± 0.002	0.704 ± 0.005	1.107 ± 0.004

**Table 5:** Effect of disperse system IRA67 resin on cultivation performance on the production of HA by *S. zooepidemicus*.

Time *	Cell Concentration (g/L)	Dissolved Oxygen (%)	Glucose (g/L)	HA (g/L)	LA (g/L)
0	0.00	98.0	50.00 ± 0.03	0.00	0.032 ± 0.24
14	2.547 ± 0.24	0.00	33.51 ± 0.23	2.763 ± 0.04	55.629 ± 0.15
15	2.325 ± 0.40	0.00	20.62 ± 0.10	2.872 ± 0.06	58.672 ± 0.09
24	2.478 ± 0.06	0.00	1.27 ± 0.78	2.713 ± 0.18	42.789 ± 0.10

* Note: For ease of sampling process (as resins were dispersed in the culture), the samples were taken at 0 h of cultivation, before (at 14 h) and after (at 15 h) the addition of the resin, and at the end of cultivation (at 24 h).

**Table 6:** Viscosity value of fermentation media of *S. zooepidemicus* in 2-L STR.

Cultivation Variable	Before Addition of IRA67 (mPa·s) *	After Addition of IRA67 (mPa·s) *	After 24 h (mPa·s) *
Control fermentation (no resin)	-	-	60.5 ± 0.05
Disperse of IRA67	60.7 ± 0.05	41.4 ± 0.1	40.0 ± 0.1
Internal column of IRA67	58.8 ± 0.1	47.0 ± 0.1	23.7 ± 0.05

* Note: The unit measurement of the viscosity is millipascal second (mPa·s).

## Data Availability

Not applicable.
